# A protein–protein interaction analysis tool for targeted cross-linking mass spectrometry

**DOI:** 10.1038/s41598-023-49663-4

**Published:** 2023-12-13

**Authors:** Jongham Park, Ahrum Son, Hyunsoo Kim

**Affiliations:** 1 Department of Bio-AI Convergence, Chungnam National University, 99 Daehak-ro, Yuseong-gu, Daejeon, 34134 Republic of Korea; 2 Department of Molecular Medicine, Scripps Research, La Jolla, CA 92037 USA; 3 Department of Convergent Bioscience and Informatics, Chungnam National University, 99 Daehak-ro, Yuseong-gu, Daejeon, 34134 Republic of Korea; 4 SCICS, 99 Daehak-ro, Yuseong-gu, Daejeon, 34134 Republic of Korea

**Keywords:** Programming language, Computational biology and bioinformatics, Bioinformatics

## Abstract

Protein networking is critical to understanding the biological functions of proteins and the underlying mechanisms of disease. However, identifying physical protein–protein interactions (PPIs) can be challenging. To gain insights into target proteins that interact with a particular disease, we need to profile all the proteins involved in the disease beforehand. Although the cross-linking mass spectrometry (XL-MS) method is a representative approach to identify physical interactions between proteins, calculating theoretical mass values for application to targeted mass spectrometry can be difficult. To address this challenge, our research team developed PPIAT, a web application that integrates information on reviewed human proteins, protein–protein interactions, cross-linkers, enzymes, and modifications. PPIAT leverages publicly accessible databases such as STRING to identify interactomes associated with target proteins. Moreover, it autonomously computes the theoretical mass value, accounting for all potential cross-linking scenarios pertinent to the application of XL-MS in SRM analysis. The outputs generated by PPIAT can be concisely represented in terms of protein interaction probabilities, complemented by findings from alternative analytical tools like Prego. These comprehensive summaries enable researchers to customize the results according to specific experimental conditions. All functions of PPIAT are available for free on the web application, making it a valuable tool for researchers studying protein–protein interactions.

## Introduction

Protein–protein interactions (PPIs) play a crucial role in the formation of protein networks and complex structures, which are essential for understanding the biological functions of proteins^1–4^. Abnormal PPIs involving endogenous proteins, proteins from pathogens, or both can lead to various human diseases, including cancer^4–6^. To understand a target protein’s interaction with a specific disease, it is necessary to profile all proteins involved in the disease beforehand. However, the profiling results may not match with an experimental target protein^7–9^. Therefore, it is essential to identify physical protein–protein interactions using various methods ^10,11^. Several web-based databases, such as STRING^12^, MINT^13^, BioGRID^14^, and IntAct^15^ provide theoretical protein–protein interaction search and contribute significantly to protein interactome profiling analysis.

Various methodologies can identify these interaction sites, including covalent labeling^16^ footprinting^17^, hydrogen–deuterium exchange mass spectrometry (HDX-MS)^18^, cross-linking mass spectrometry (XL-MS or CX-MS or CL-MS)^11,19–26^, ion-mobility MS^27^, and native MS^10,28^. Among these methods, XL-MS has become a powerful approach for mapping protein–protein interactions over several decades^23^. However, its effectiveness has long been impeded by three primary obstacles: (1) complex tandem mass spectrometry (MS/MS or MS2) fragmentation of cross-linked peptides; (2) low abundance of cross-linked peptides in complex peptide mixtures; (3) heterogeneity of cross-linked products^10^. Furthermore, calculating all possible cases and their mass values by considering the site, charge, and modification where interactions between proteins occur can be challenging. The first hurdle makes accurate identification of cross-linked peptides and unambiguous assignment of cross-linked sites challenging, while the second and third hurdles hinder effective MS detection of cross-linked peptides^4^. The fourth hurdle becomes significantly hinders the efficiency of research. Selected reaction monitoring (SRM) method is used to identify PPIs perform based on MS/MS values obtained through profiling. However, MS/MS fragmentation of cross-linked peptides is typically convoluted and unpredictable. Therefore, to identify the site of the predicted theoretical actual interaction occurs and calculate their mass values, we need to compare experimental MS/MS spectra against a computed library of theoretical spectra. This process of calculating mass values must not only consider all cases of theoretical interaction but also the properties of cross-linker, charge of peptide/fragmented peptide ion, and modification. Finally, several tools can help analyze XL-MS, such as SRMcollider^29^, Prego^30^, and X-Link Transition Calculator^31^ in Skyline^32^. SRMcollider is software that predicts interference probability between target transitions^29^. Prego is software that predicts high-responding peptides for SRM experiments^30^. The X-Link Transition Calculator is software that calculates cross-linked mass values^31^. However, these tools are unable to search theoretical protein interactors for the target protein, and it must be known which sites are actual interact and modified. In this respect, the development of analysis tools for XL-MS is still needed.

We proposed PPIAT, a targeted mass spectrometry-based protein–protein interaction analysis tool. PPIAT is a web-based analysis tool that can search interaction information about human proteins and calculate mass values given the properties of cross-linkers, enzymes, charges, and modifications. We expect PPIAT to improve experimental efficiency and become a significant contributor to theoretical information for XL-MS analysis.

## Materials and methods

### Overview of PPIAT

**Figure 1:**
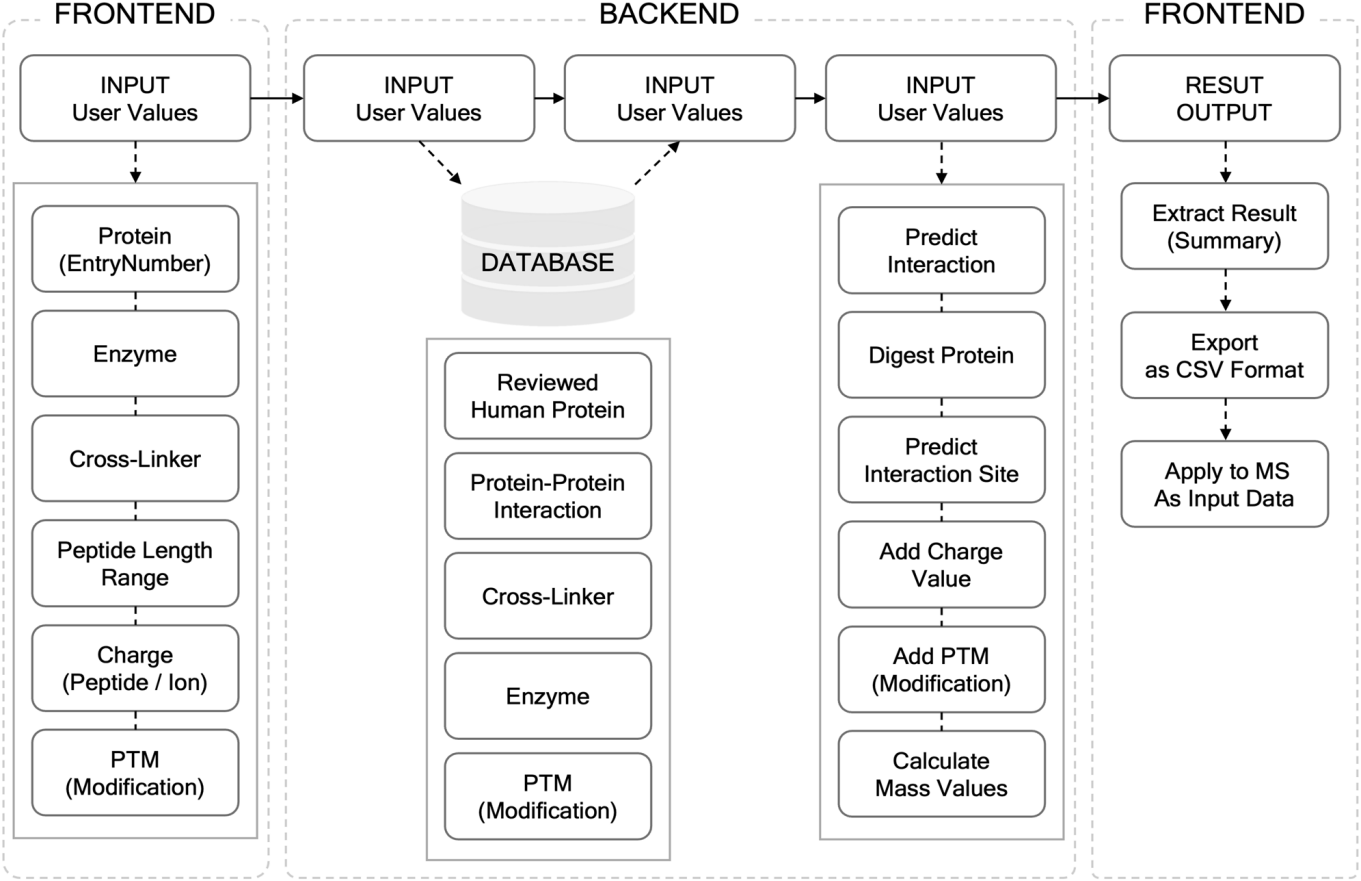
Workflow of PPIAT. The figure illustrates the flow of PPIAT, with input Information for searching protein–protein interaction (PPIs) at the front-end. Data is queried from the database based on the input information, and PPIs and mass values are calculated according to the input conditions. All calculated data is presented at the front-end, and the results can be exported in CSV format. The exported data can used as input for MS/MS analysis.

PPIAT was developed using XAMPP, CI3 (Codeignator3), and CSS (Cascading Style Sheet). XAMPP is a software package that includes the Apache web server, PHP (Hypertext Preprocessor), and MySQL, which means that PPIAT runs on the Apache server and relies on the MySQL database. The software offers three main functions. First, it allows users to search for human proteins by utilizing information from UniProt’s reviewed human proteins database^24,33^. Second, it provides users with the ability to search for information on commercially available cross-linkers, including their name, binding site, cleavability^25^, spacer arm length, and mass values, which are differentiated between non-cleavage and cleavage mass values. Third, PPIAT can predict protein–protein interactions for the user’s target protein and calculate their mass values based on input parameters such as target protein, cross-linker, enzyme, peptide length range, peptide charge, ion charge, and modifications specified on the search page. Finally, the results obtained from PPIAT can be summarized and extracted based on the probability score of protein interaction in the derived results and other analysis tools such as Prego^30^. With these functions, PPIAT serves as a valuable for targeted mass spectrometry-based protein interactor analysis in XL-MS. (see Fig. 1) To aid users in effectively utilizing the tool, a comprehensive tutorial is accessible at: http://ppiat.cnu.ac.kr/userguide.

### Database construction

PPIAT is built on a comprehensive database that integrates data from various sources, including reviewed human proteins, protein–protein interactions, cross-linkers, enzymes, and modifications. The database contains 20,386 reviewed human protein entries (43,277 including isoforms), approximately 20 billion interaction data, and 83 cross-linker entries. These data were collected from reputable sources such as UniProt^33^, STRING^12^, and publicly published white papers. PPIAT employs the STRING database to identify interacting proteomes associated with target proteins. The databases for PPIs and reviewed human proteins, provided respectively by STRING and UniProt, are initially distinct in format. PPIAT enhances its predictive capability by integrating these two databases into a cohesive format for PPI search. The database forms the backbone of the PPIAT, enabling the efficient and accurate prediction of protein–protein interactions and the calculation of their respective mass values.

### User guide about PPIAT

**Figure 2:**
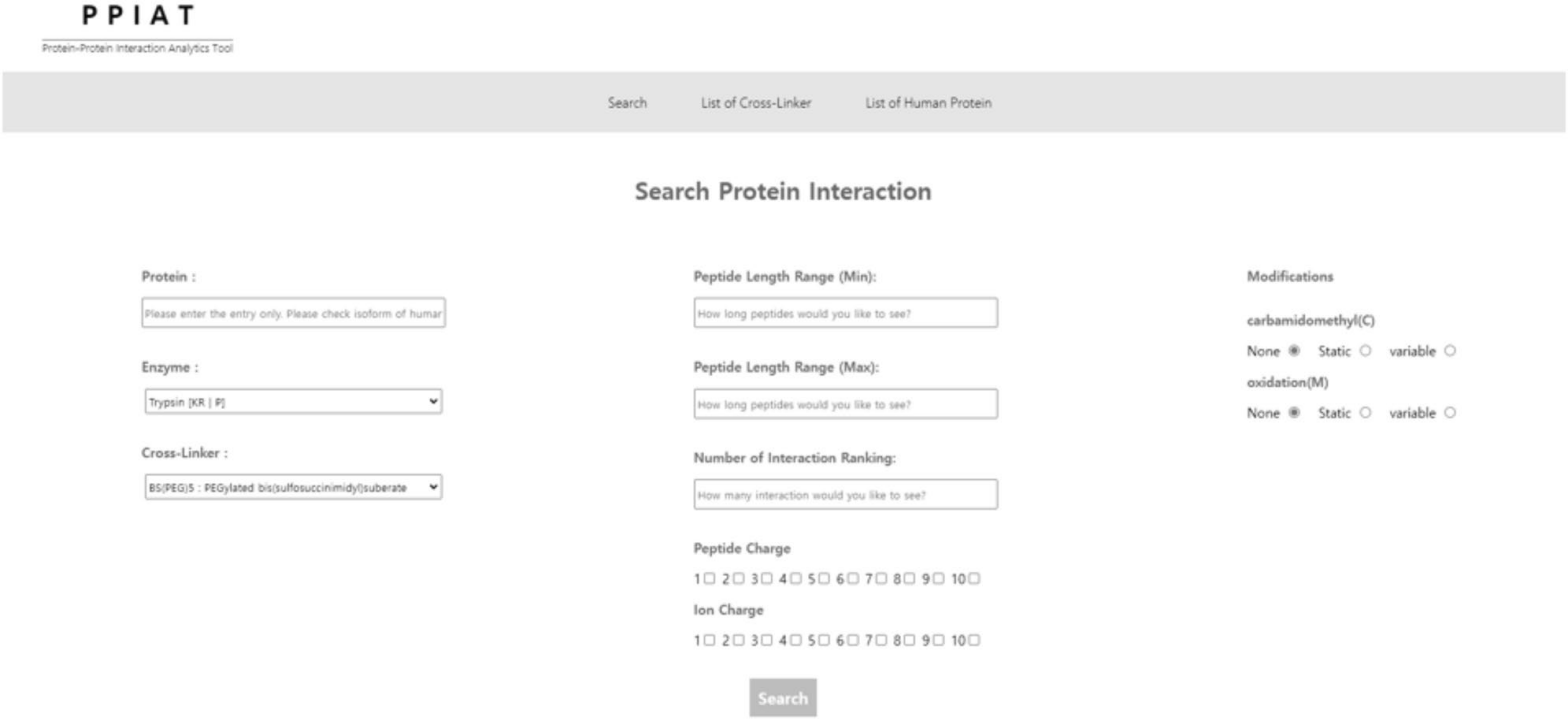
PPIAT search page. This search page of PPIAT requires users to input specific information for searching protein–protein interactions (PPIs) and their mass values. Required information includes the entry number of the target protein, the enzyme used for protein digestion, the cross-linker utilized for crosslinking, the range of peptide length, the ranking of protein–protein interactions, the charges of the peptide and ion, and any predicted protein modifications.

PPIAT offers a user-friendly interface, allowing for the input of various parameters, such as (1) the entry number of the desired target protein (e.g., P04439, Q6ZWK4), (2) the enzyme used for protein digestion, (3) the cross-linker employed in XL-MS^11,19–26^ for identifying protein–protein interactions, (4) the preferred peptide length range for MS/MS analysis, (5) the peptide and fragmented peptide ion charges, and (6) any anticipated modifications in the protein–protein interactions (refer to Fig. 2).The information of reviewed human proteins available at UniProt^33^ comprises several columns, including ID, name, organism, entry number, entry name, and sequence ID. The entry number and entry name information can be utilized to search for protein–protein interactions in resources such as STRING^12^.

Proteins are differentiated between canonical and isoform. Isoform refers to a member of a set of highly similar proteins that originate from a single gene or gene family and are the result of genetic differences. Although isoforms have the same or similar function, they do not have an identical sequence and therefore have different mass values^34^. To address this issue, PPIAT divides the same entry name and protein name between canonical and isoform using a dash (“- “) in the entry number. However, while the values of the string column at UniProt^33^ and values of protein A and protein B at STRING^12^ are linked to each other (e.g., format of 9606.ENSP00000379873), they are not completely matched, and the canonical and isoform use the same value. It should be noted that PPIAT does not differentiate between canonical forms and isoforms of a target protein based solely on the protein A and B values from STRING^12^. For distinguishing between these forms, PPIAT utilizes the entry number when searching for a target protein (e.g., P02649, P06727).

Users can select either trypsin or chymotrypsin as the enzyme for protein digestion, depending on the properties of the digestion site and exceptions. In addition, users can choose from a list of 83 commercialized cross-linkers to identify interactions between the target protein and other proteins based on the properties of the cross-linker binding site and cleavability. (see Data S1) Finally, by inputting the peptide length range, the number of interaction rankings, the charge of the peptide and fragmented peptide ion, and any modifications predicted to occur in the protein–protein interactions. The peptide length range refers to the maximum and minimum peptide lengths that the user wants to identify at the interaction level, and the number of interaction rankings refers to the number of interactable proteins that the user wants to identify. The input values for peptide charge and fragmented peptide ion charge considered for MS/MS analysis allow for overlapping selection. There are two types of modifications, static and valuable, which are used to calculate mass values, and they also allow for overlapping selection. PPIAT is designed to identify interactions with target proteins and automatically compute the totality of actual interaction cases, along with their corresponding masses resulting from cross-linking at both peptide and ionic levels. This is done while taking into consideration the charge of the peptide and fragmented peptide ion, properties of the cross-linker and enzyme, as well as any modifications.

## Result

The XL-MS workflow can be divided into several steps. Firstly, a target protein complex is cross-linked in solution and digested with trypsin into peptides. Secondly, the peptides are analyzed by liquid chromatography coupled with mass spectrometry (LC–MS/MS) to obtain precursor masses and fragment masses for cross-linked peptides. Thirdly, the fragmentation spectra of all peptides are subjected to database searching to identify cross-linked peptides^20^. This procedure involves searching for the binding site of the cross-linker in each sequence of the precursor ion digested by the enzyme and the fragmented ion generated during SRM analysis. Considering the varying mass of the cross-linked interactor depending on the cross-linker's characteristics, PPIAT consults a database encompassing information about the cross-linker's properties, such as its cleavability. If there are amino acids that can bind to the cross-linker, PPIAT calculates the mass, accounting for the cross-linker's properties and all potential cross-linking types. Finally, the output of PPIAT can be summarized and extracted based on the probability score of protein interaction in the derived results and other analysis tools such as Prego^30^. This output can be used as input data for targeted mass spectrometry analysis. The combined score is divided into four groups based on the confidence level: highest confidence (> 0.9), high confidence (> 0.7), medium confidence (> 0.4), and low confidence (> 0.15). The analysis program Prego provides a list of predicted high-responding peptides for SRM experiments^30^. PPIAT stands out for its advanced mass calculation capabilities, vital for integrating XL-MS studies with targeted mass spectrometry techniques such as SRM (Selected Reaction Monitoring) and PRM (Parallel Reaction Monitoring). It enables the generation of comprehensive mass spectral libraries from results obtained through DDA (Data-Dependent Acquisition) or DIA (Data-Independent Acquisition) analyses, effectively targeting specific proteins. It also facilitates conducting experiments with crosslinkers on peptide sequences predicted by Prego^30^.

The PPIAT software outputs results that can be utilized as input for SRM analysis. This software searches for PPIs and calculates their mass values. It generates separate tables for identified PPIs and predicts interaction information, indicating which proteins are interacting with the target protein based on a combined score. Additionally, another table provides printed information on the theoretical actual interaction, including combined score, the protein A’ peptide, cross linker, protein B’ peptide, precursor charge, precursor *m/z*, protein A’ ion, protein A’ ion type, protein B’ ion, protein B’ ion type, product charge, and product *m/z*.

**Figure 3:**
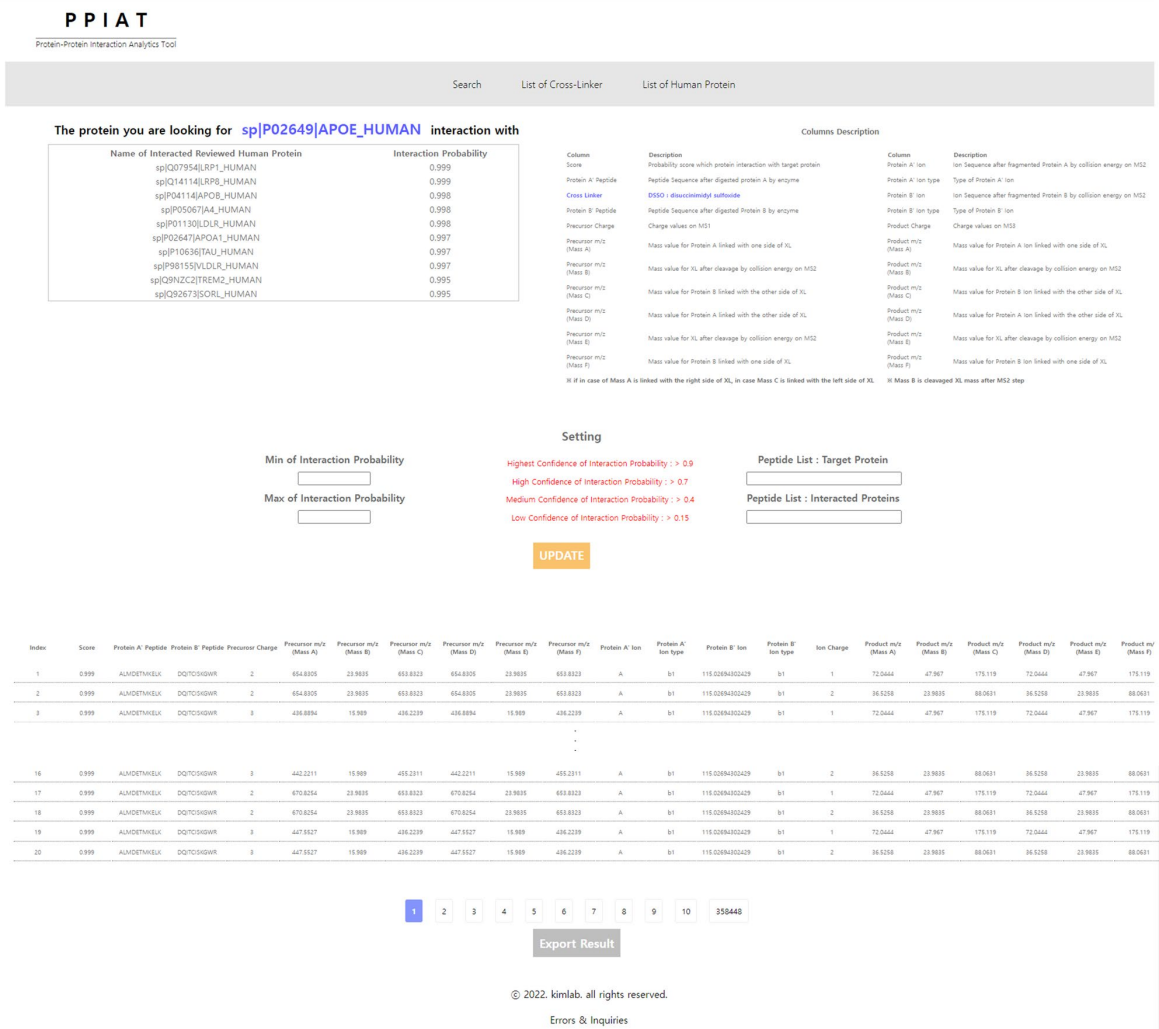
Result page of PPIAT, which consists of two main sections. The first section identifies the protein–protein interactions (PPIs) and predicts the interacting proteins for the target protein, based on a combined score from the STRING database. The second section calculates the mass values for the PPIs, taking into consideration the conditions for cross-linking mass spectrometry (XL-MS).

After searching for the target protein using PPIAT, the software generates a list of theoretical PPIs for the target protein, along with the number of actual interactions that occur in the interactome and their corresponding mass values. The theoretical PPIs list predicts which proteins interact with the target protein and assigns scores based on the STRING database^12^. In each column of the tables, “Protein A” refers to the target protein that was inputted, while “Protein B” refers to the proteins that interact with the target protein in both peptide and ion levels. The precursor m/z and product m/z columns are divided into the mass value of protein A linked with one side of the cross linker, the cross-linker mass after cleavage, and protein B linked with the other side of cross linker. If a modification is expected to occur statically, the fluctuating mass values are added. However, if the modification is expected to occur variably, the results are printed as two lines—one with the change in mass values due to the modification and one without. Figure 3 provides an example of the output format.

Cross-linking mass spectrometry (XL-MS) involves performing a mass spectrometry-based analysis on cross-linked complexes that have been digested by enzymes. When using PPIAT, the software calculates the mass values of the digested cross-linked protein complexes at the peptide and fragmented peptide ion levels. However, it does not consider the total mass of the cross-linked complex. To take into account all other products of the cross-linking reaction between Protein A and Protein B, it is necessary to consider four possibilities at the peptide and fragmented peptide ion levels: (1) cross-linked between Protein A and Protein B (cross-linking or interpeptide); (2) only cross-linked with Protein A, not cross-linked to Protein B (loop-link or intrapeptide); (3) only cross-linked with Protein B, not cross-linked to Protein A (loop-link or intrapeptide); and (4) non-cross-linked between Protein A and Protein B. (mono-link or dead-end)^20,21^ In addition, when conducting XL-MS experiments, it is possible to encounter situations where interactions occur on one side, and the other side of the cross-linker may interact with Protein A and Protein B, respectively. To obtain more accurate data of the protein–protein interactions, these possibilities must be considered.

Cross-linkers are typically classified into two categories: cleavable and non-cleavable cross-linkers^35,36^. When using a cleavable cross-linker, it is important to consider its properties, such as the cleavage site and the type of cleavage that occurs (e.g., symmetric or asymmetric) ^35,36^. If a cleavable cross-linker is used and is cleaved symmetrically, the same mass values should be added to Protein A and Protein B, respectively. However, if the cross-linker is cleaved asymmetrically, different mass values must be added. It is important to consider these factors when using a cleavable cross-linker to obtain accurate results. All calculated mass values can be exported to CSV format and used as input data for MS/MS analysis, which can provide further insights into the protein–protein interactions.

We utilized the PPIAT tool to explore potential PPIs involving APOE, a major biomarker for Alzheimer’s disease^37–45^. Our search was performed under the following conditions: APOE(UniProt ID: P02649), trypsin digestion, cross-linker DSSO, top 10 interactions, peptide length of 9, peptide charge of 2 and 3, ion charge of 1 and 2, and modifications of carbamidomethylation(static) and oxidation(variable). Ten proteins were identified by PPIAT, including LRP8(UniProt ID: Q14114, score:0.999), LRP1(UniProt ID: Q07954, score:0.999), A4(APP, UniProt ID: P05067, score:0.998), APOB(UniProt ID: P04114, score:0.998), LDLR(UniProt ID: P01130, score:0.998), APOA1(UniProt ID: P02647, score:0.997), VLDLR(UniProt ID: P98155, score:0.997), TAU(MAPT, UniProt ID: P10636, score:0.997), SORL(UniProt ID: Q92673, score:0.995), and TREM2(UniProt ID: Q9NZC2, score: 0.995) (Table 1), along with their theoretical information in XL (see Data S2 and S3). We verified that the PPIs output information matched the search results from STRING. Additionally, we confirmed the mass shift was consistent with the cross-linker DSSO by comparing the mass values using Skyline software (see Data S2 and S3).

**Table 1:** APOE E4 PPIs search from PPIAT.

Rank	Protein name	Interaction probability
1	sp|Q07954|LRP1_HUMAN	0.999
2	sp|Q14114|LRP8_HUMAN	0.999
3	sp|P04114|APOB_HUMAN	0.998
4	sp|P05067|A4_HUMAN	0.998
5	sp|P01130|LDLR_HUMAN	0.998
6	sp|P02647|APOA1_HUMAN	0.997
7	sp|P10636|TAU_HUMAN	0.997
8	sp|P98155|VLDLR_HUMAN	0.997
9	sp|Q9NZC2|TREM2_HUMAN	0.995
10	sp|Q92673|SORL_HUMAN	0.995

Top 10 list of proteins interaction with APOE E4 protein. The results are matched with results on STRING.

## Discussion

XL-MS is a widely used method for identifying protein interactions and elucidating protein structures in three dimensions. In recent years, significant technological advancements in XL-MS studies have propelled the field of proteomics forward. However, there are still several limitations that need to be overcome, such as the need for better strategies for cross-linking. Unfortunately, there is currently no available solution or analysis tool that can accurately calculate the mass values of theoretical interactions required for SRM analysis. To address this challenge, our research team has developed PPIAT, a tool that can search for protein–protein interactions with target proteins, identify theoretical interactions that occur, and calculate their mass values at the peptide and fragmented peptide ion levels. With PPIAT as input data, mass values can be accurately calculated. This new tool provides an important advancement in the field of proteomics, helping researchers to overcome the challenges of XL-MS and improve their ability to identify protein–protein interactions.

Overall, the development of PPIAT represents a significant advancement in XL-MS studies and has the potential to enhance our understanding of protein–protein interactions. Although strategies such as the development of various cross-linkers have been employed in XL-MS, a lack of solutions or analysis tools for accurately calculating the mass values of theoretical interactions required for MS analysis still exists. To address this issue, several software tools have been developed, including SRMcollider^29^, Prego^30^, and X-Link Transition Calculator^31^ in Skyline. However, these tools are not integrated with the platform used to search for theoretical PPIs, and the protein modification, binding site, and mass value of the cross-linker must be calculated and entered directly for XL-MS.

While PPIAT is a valuable tool for XL-MS analysis, it has some limitations that need to be addressed. Firstly, it does not cover all the products of the cross-linker, nor does it provide information about the enzyme or modification. Additionally, the output generated by PPIAT is only theoretical and requires experimental verification to confirm the results. To address these limitations, our team has built a database that includes information on 83 different cross-linkers, two types of enzymes (trypsin and chymotrypsin)^20^, and the most commonly occurring modifications (oxidation and carbamidomethylation). While these updates are a step forward, we will continue to improve and update the database regularly to ensure that it remains a useful resource for the scientific community.

The field of cross-linkers has seen a wide range of types with unique properties, including chemical cross-linkers and Photo/MS-cleavable cross-linkers^35,36^^46^, which has been studied and commercialized. Additionally, researchers often synthesize their own cross-linkers with novel characteristics, such as BMSO, DBB, DHSO, and SDASO, for their experiments^47–51^. Although these synthetic cross-linkers are not currently included in the PPIAT database, an editing function will be added to the software to allow users to input information on these cross-linkers when searching for target proteins.

The PPIAT database is continuously updated with reviewed information on human proteins and protein–protein interactions. While the current version of the database has been built using PPI information from STRING^12^ only, our plan is to expand it by integrating with other databases such as MINT^13^, BioGRID^14^, and IntAct^15^ for PPI searching. The output generated by PPIAT can be downloaded in CSV format and used as input data for SRM analysis.

This analysis tool was developed on i9-10940X CPU, 128 GB RAM, Window server system. Researchers can identify PPIs up to the following conditions: (1) protein: AP02649 (APOE protein), (2) enzyme: trypsin, (3) cross-linker: DSSO, (4) peptide length range (min): 6, (5) peptide length range (max):10, number of interaction ranking: 10, (6) precursor charge: 2, 3, (7) product charge: 1, 2, (8) modifications: carbamidomethyl (C): static, oxidation (M): variable. The current analysis conditions are limited. We plan to make a server system that allows search conditions with at least 10 interacting proteins for the target protein and a peptide length ranging from 6 to 20.

## Conclusions

In conclusion, this paper introduces PPIAT, a web-based open platform designed for the analysis of XL-MS data. PPIAT provides a solution for the search of protein–protein interactions involving target proteins and calculation of the mass values of all theoretical interaction cases between proteins at both peptide and fragmented peptide ion levels, overcoming a significant obstacle in XL-MS analysis. We expect that this tool will be widely adopted by researchers in proteomics and bioinformatics, and we welcome contributions to its development. By continuously updating and refining the database, we aim to address the current limitations and improve the accuracy and efficiency of XL-MS analysis.

### Supplementary Information

Supplementary Information.

## Data Availability

PPIAT is freely available at ppiat.cnu.ac.kr/. All data utilized in this study were sourced from esteemed databases and publications. Specifically, the human protein data employed for targeted protein searches and the protein–protein interaction data for interaction analyses were acquired from the UniProt and STRING databases, respectively. These repositories are renowned for their rigorous maintenance and reliability. Additionally, the cross-linker data, utilized by the Protein–Protein Interaction Analysis Tool (PPIAT), was sourced from peer-reviewed white papers and is accessible in Supplementary Data 1. The novel data generated in this study can be found in Supplementary Data 3. We ensure full accessibility of these data; researchers can freely utilize all data processed through PPIAT, which are exportable in CSV format directly from the results page.

## Code availability

The source code for PPIAT is available on GitHub at https://github.com/kimlab-cnu.
