# Supplementary material for: A protein–protein interaction analysis tool for targeted cross-linking mass spectrometry
Source: Sci Rep. 2023 Dec 13;13:22103. doi: 10.1038/s41598-023-49663-4 (PMC10719354; doi:10.1038/s41598-023-49663-4)
Supplement: Supplementary file 1 — Supplementary Information. [file 41598_2023_49663_MOESM1_ESM.zip › Data_S1_List_of_Cross_Linker_Editor_Only.docx]

**Data S1.List of Cross linkers in PPIAT : Dashed Lines Indicate Cleavage Sites**

| Name | Cleavability | Formula | Monoisotopic | Formula  (Cleavaged) | Monoisotopic  (Cleavaged) | Structure |
| --- | --- | --- | --- | --- | --- | --- |
| BS(NHS)PEG5 | No | C_22_H_32_N_2_O_13_ | 532.499 | C_22_H_32_N_2_O_13_ | 532.499 | 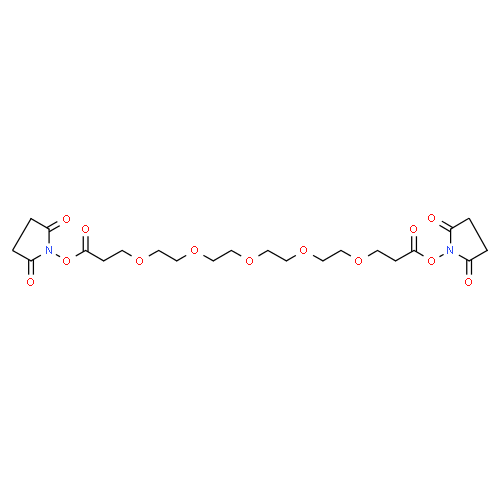 |
| BS(PEG)9 | No | C_30_H_48_N_2_O_17_ | 708.711 | C_30_H_48_N_2_O_17_ | 708.711 | 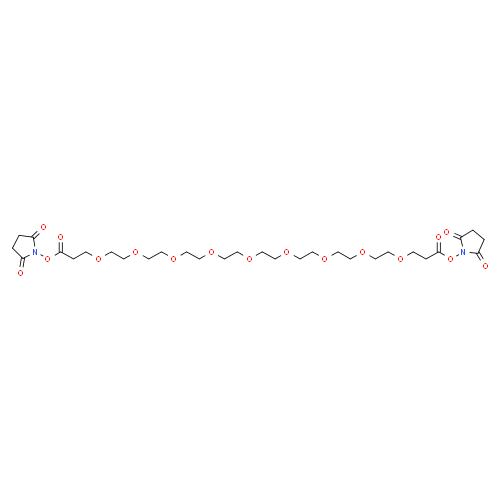 |
| BS2G-d0 | No | C_13_H_12_N_2_Na_2_O_14_S_2_ | 530.351 | C_13_H_12_N_2_Na_2_O_14_S_2_ | 530.351 | 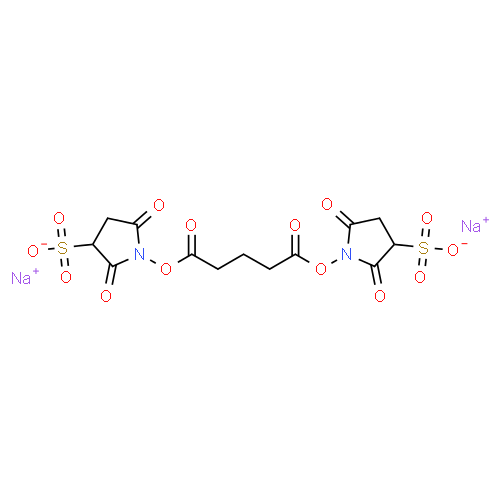 |
| BS2G-d4 | No | C_13_H_8_D_4_N_2_Na_2_O_14_S_2_ | 534.383 | C_13_H_8_D_4_N_2_Na_2_O_14_S_2_ | 534.383 | 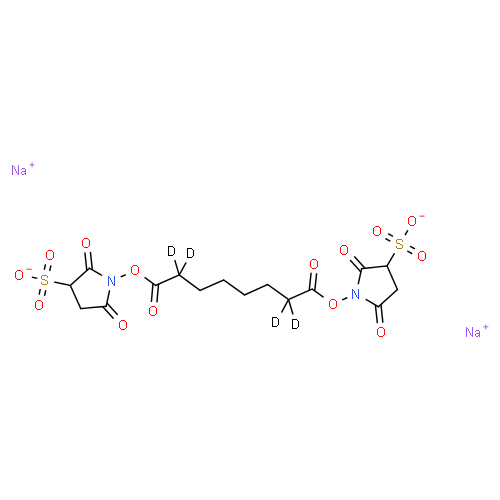 |
| BS3 | No | C_16_H_18_N_2_O_14_S_2_Na_2_ | 572.432 | C_16_H_18_N_2_O_14_S_2_Na_2_ | 572.432 | 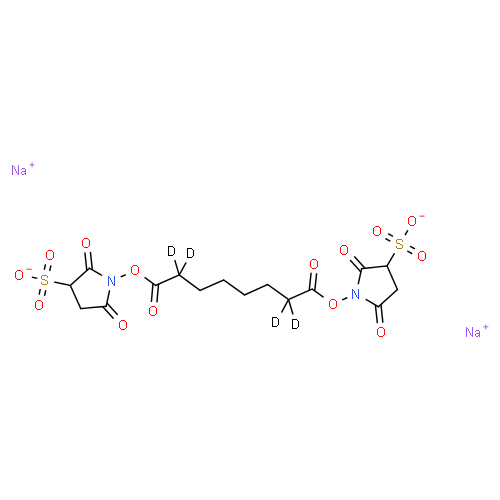 |
| BS3-d4 | No | C_16_H_14_D_4_N_2_Na_2_O_14_S_2_ | 576.464 | C_16_H_14_D_4_N_2_Na_2_O_14_S_2_ | 576.464 | 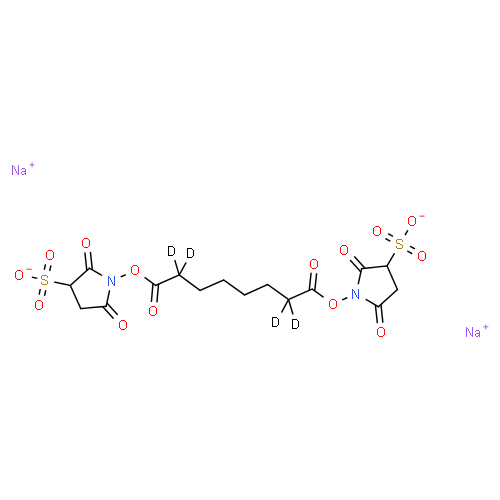 |
| DFDNB | No | C_6_H_2_F_2_N_2_O_4_ | 204.088 | C_6_H_2_F_2_N_2_O_4_ | 204.088 | 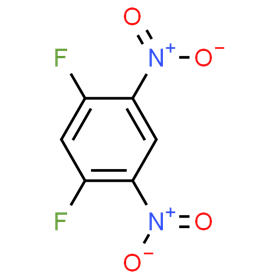 |
| DMP | No | C_9_H_20_Cl_2_N_2_O_2_ | 259.177 | C_9_H_20_Cl_2_N_2_O_2_ | 259.177 | 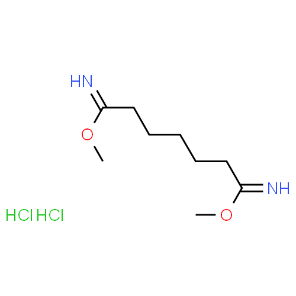 |
| DMS | No | C_10_H_22_N_2_O_2_Cl_2_ | 273.204 | C_10_H_22_N_2_O_2_Cl_2_ | 273.204 | 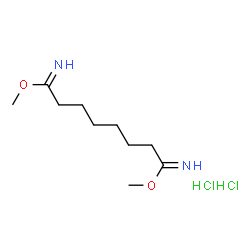 |
| DSG | No | C_13_H_14_N_2_O_8_ | 326.261 | C_13_H_14_N_2_O_8_ | 326.261 | 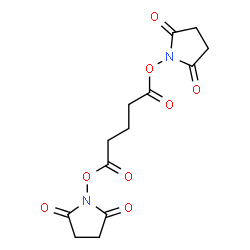 |
| DSS | No | C_16_H_20_N_2_O_8_ | 368.342 | C_16_H_20_N_2_O_8_ | 368.342 | 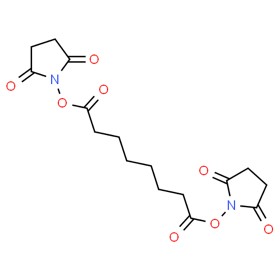 |
| TSAT | No | C_18_H_18_N_4_O_12_ | 482.358 | C_18_H_18_N_4_O_12_ | 482.358 | 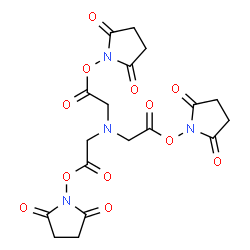 |
| DSP | Yes | C_14_H_16_N_2_O_8_S_2_ | 404.420 | C_7_H_8_NO_4_S  C_7_H_8_NO_4_S | 202.210  202.210 | 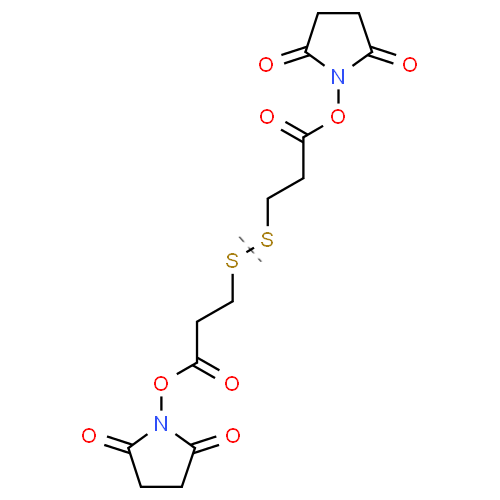 |
| DTBP | Yes | C_8_H_18_Cl_2_N_2_O_2_S_2_ | 309.282 | C_4_H_9_ClNOS  C_4_H_9_ClNOS | 154.641  154.641 | 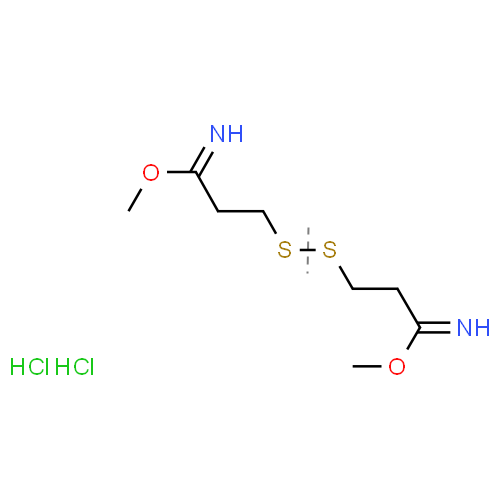 |
| DTSSP | Yes | C_14_H_14_N_2_Na_2_O_14_S_4_ | 608.510 | C_7_H_7_NNaO_7_S_2_  C_7_H_7_NNaO_7_S_2_ | 304.255  304.255 | 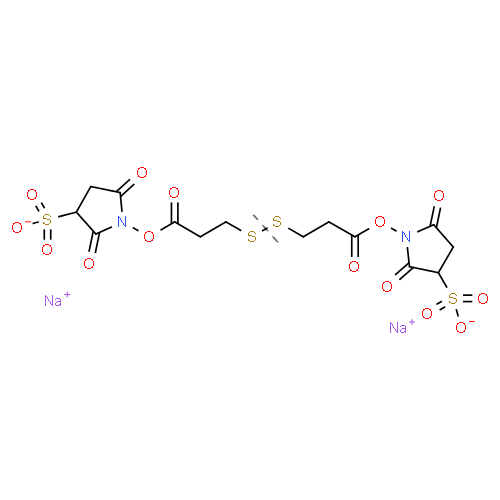 |
| EGS | Yes | C_18_H_20_N_2_O_12_ | 456.360 | C_8_H_8_NO_5_  C_2_H_4_O_2_  C_8_H_8_NO_5_ | 198.154  60.052  198.154 | 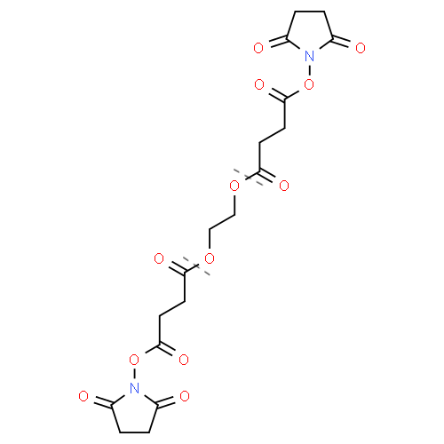 |
| Sulfo-EGS | Yes | C_18_H_18_N_2_Na_2_O_18_S_2_ | 660.450 | C_8_H_7_NNaO_8_S  C_2_H_4_O_2_  C_8_H_7_NaO_8_S | 300.199  60.052  300.199 | 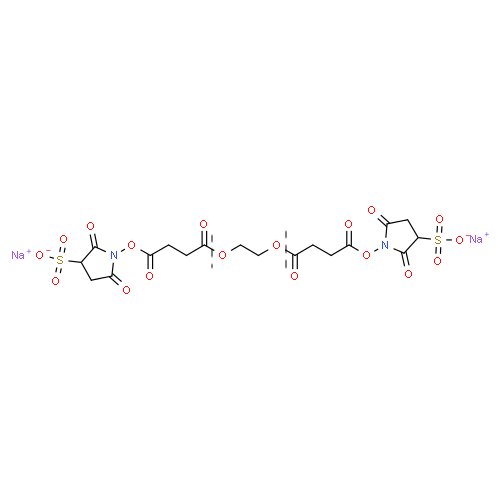 |
| DSBU | Yes | C_17_H_22_N_4_O_9_ | 426.382 | C_8_H_11_N_2_O_4_  CO  C_8_H_11_N_2_O_4_ | 199.186  28.010  199.186 | 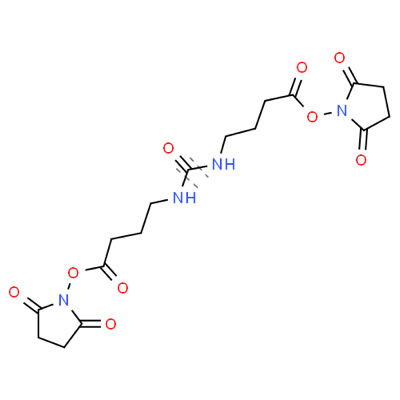 |
| DSSO | Yes | C_14_H_16_N_2_O_9_S | 388.353 | C_7_H_8_NO_4_  SO  C_7_H_8_NO_4_ | 170.144  48.065  170.144 | 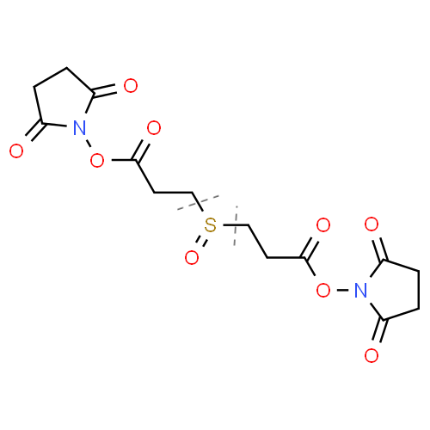 |
| DST | Yes | C_12_H_12_N_2_O_10_ | 344.232 | C_6_H_6_NO_5_  C_6_H_6_NO_5_ | 172.116  172.116 | 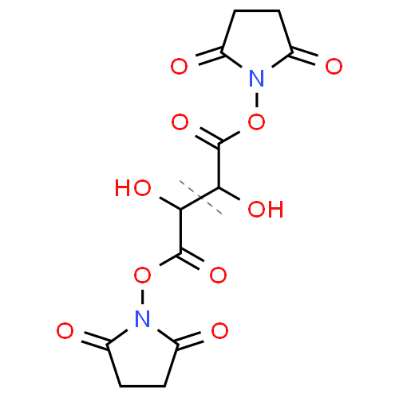 |
| NHS-Azide | No | C_6_H_6_N_4_O_4_ | 198.138 | C_6_H_6_N_4_O_4_ | 198.138 | 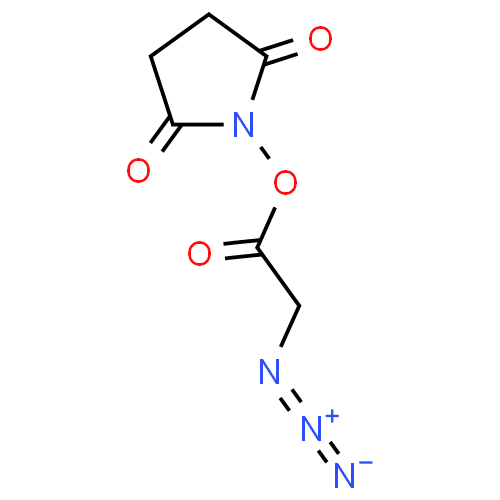 |
| NHS-PEG4-Azide | No | C_15_H_24_N_4_O_8_ | 388.377 | C_15_H_24_N_4_O_8_ | 388.377 | 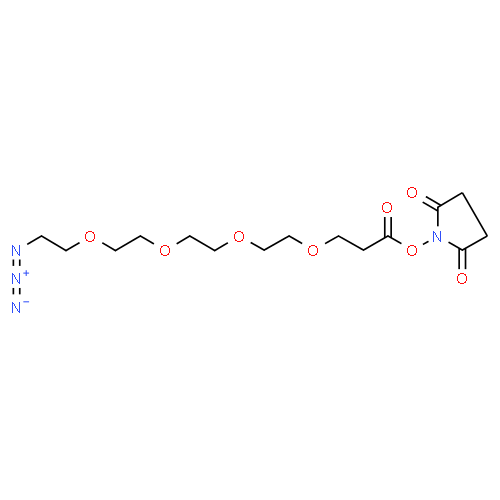 |
| NHS-Phosphine | No | C_25_H_20_NO_6_P | 461.410 | C_25_H_20_NO_6_P | 461.410 | 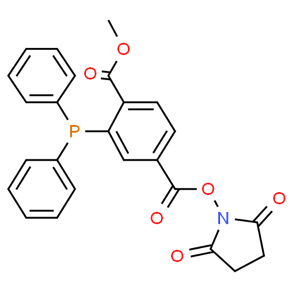 |
| EDC | No | C_8_H_18_ClN_3_ | 191.706 | C_8_H_18_ClN_3_ | 191.706 | 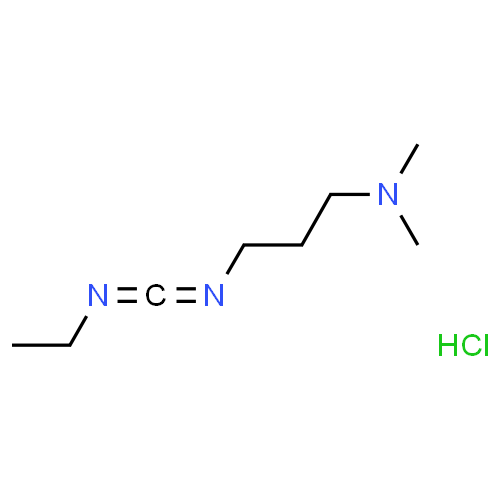 |
| NHS | No | C_4_H_5_NO_3_ | 115.088 | C_4_H_5_NO_3_ | 115.088 | 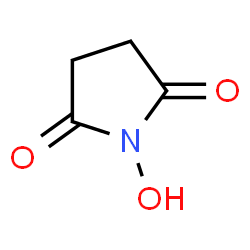 |
| Sulfo-NHS | No | C_4_H_4_NNaO_6_S | 217.133 | C_4_H_4_NNaO_6_S | 217.133 | 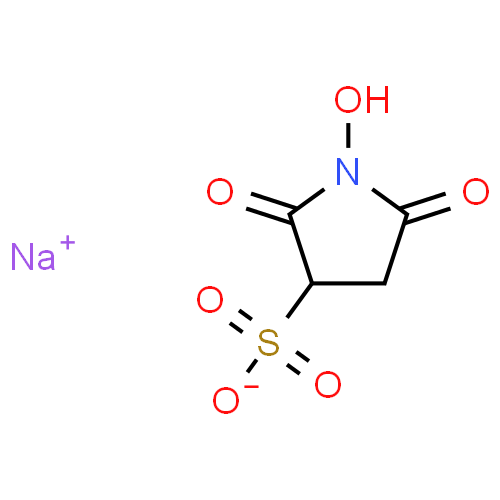 |
| LC-SDA | No | C_15_H_22_N_4_O_5_ | 338.364 | C_15_H_22_N_4_O_5_ | 338.364 | 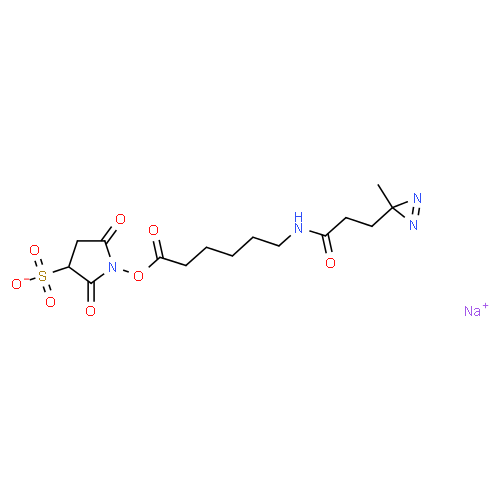 |
| SDA  (NHS-Diazirine) | No | C_9_H_11_N_3_O_4_ | 225.204 | C_9_H_11_N_3_O_4_ | 225.204 | 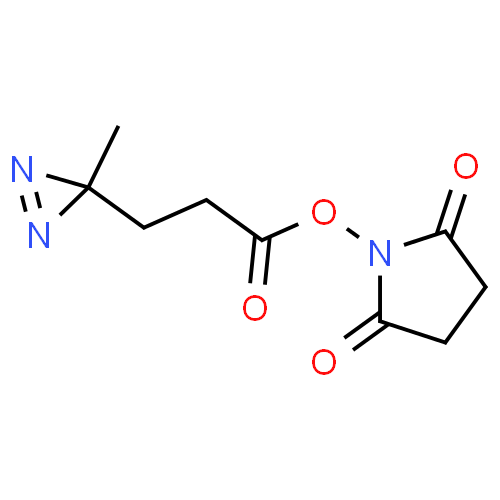 |
| SDAD  (NHS-SS-Diazirine) | Yes | C_14_H_20_N_4_O_5_S_2_ | 388.469 | C_7_H_12_N_3_OS  C_7_H_8_NO_4_S | 186.259  202.210 | 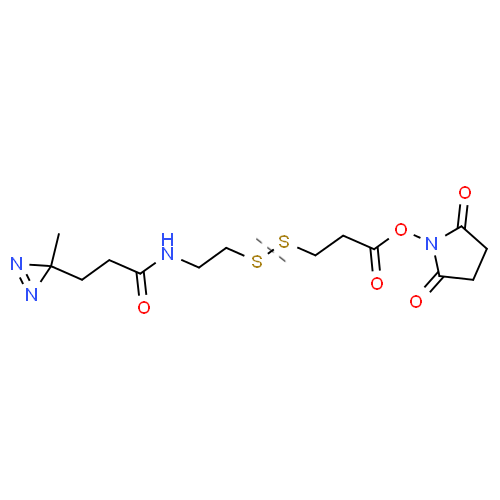 |
| SPB | No | C_19_H_15_NO_8_ | 385.328 | C_19_H_15_NO_8_ | 385.328 | 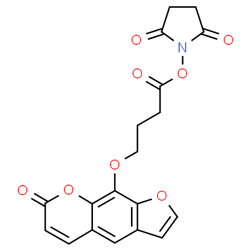 |
| Sulfo-LC-SDA | No | C_15_H_21_N_4_NaO_8_S | 440.409 | C_15_H_21_N_4_NaO_8_S | 440.409 | 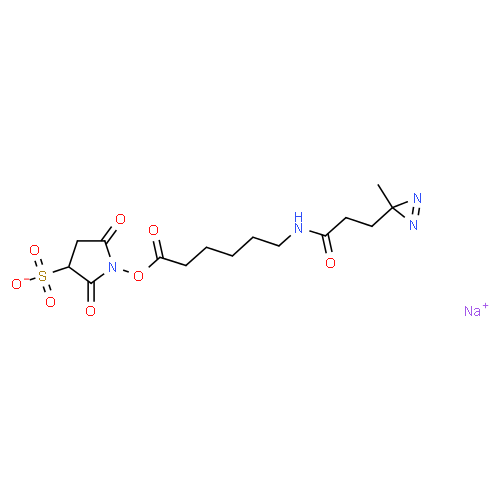 |
| Sulfo-SANPAH | No | C_16_H_17_O_9_N_6_NaS | 492.401 | C_16_H_17_O_9_N_6_NaS | 492.401 | 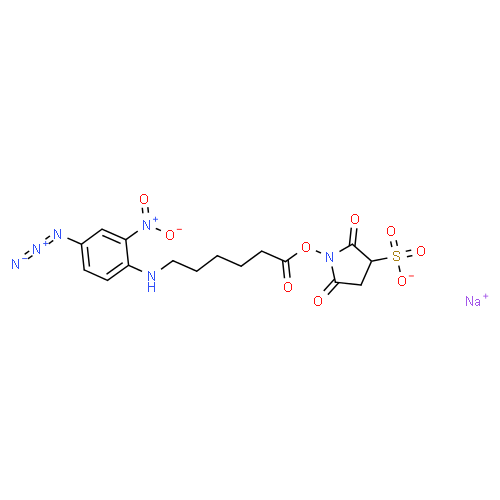 |
| Sulfo-SBED | Yes | C_32_H_42_N_9_NaO_11_S_4_ | 878.986 | C_25_H_35_N_8_O_4_S_2_  C_7_H_7_NNaO_7_S_2_ | 588.738  290.248 | 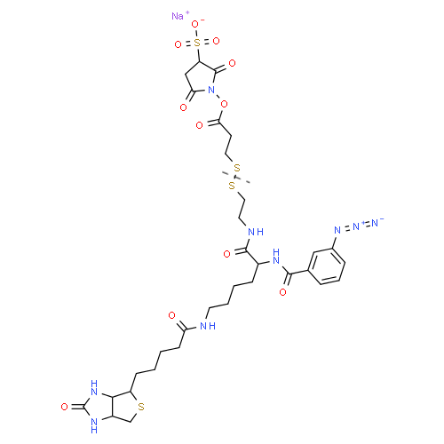 |
| Sulfo-SDA | No | C_9_H_10_N_3_Na_7_S | 327.249 | C_9_H_10_N_3_Na_7_S | 327.249 | 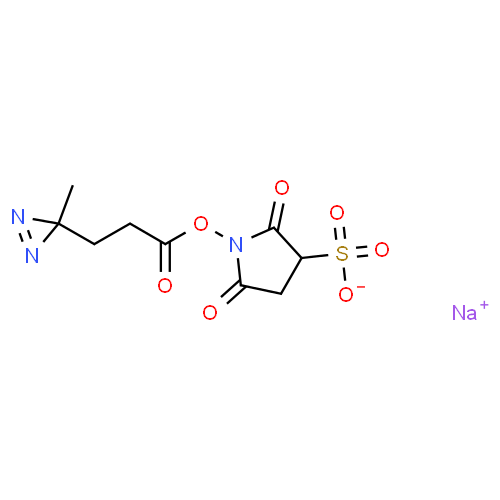 |
| Sulfo-SDAD | Yes | C_14_H_19_N_4_NaO_8_S_3_ | 490.514 | C_7_H_7_NNaO_7_S_2_  C_7_H_12_N_3_OS | 304.255  186.259 | 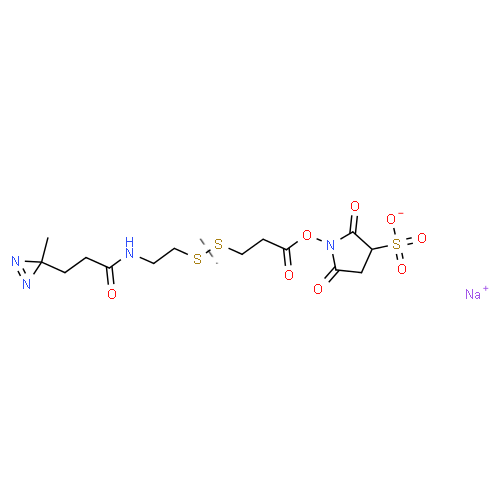 |
| AMAS | No | C_10_H_8_N_2_O_6_ | 252.182 | C_10_H_8_N_2_O_6_ | 252.182 | 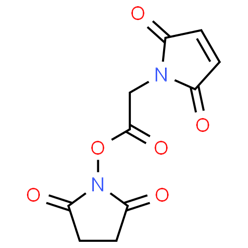 |
| BMPS | No | C_11_H_10_N_2_O_6_ | 266.209 | C_11_H_10_N_2_O_6_ | 266.209 | 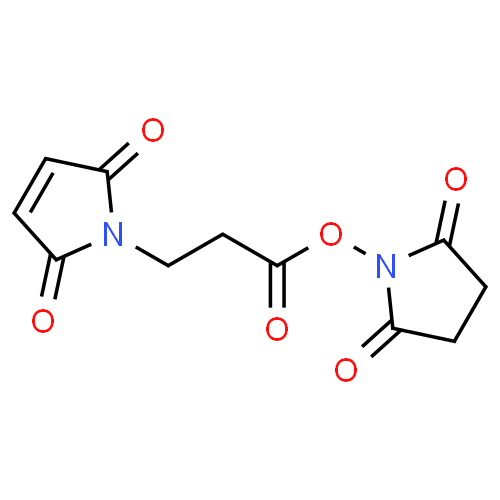 |
| EMCA | No | C_10_H_13_NO_4_ | 211.217 | C_10_H_13_NO_4_ | 211.217 | 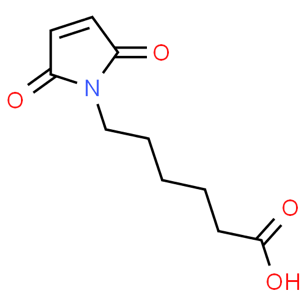 |
| EMCS | No | C_14_H_16_N_2_O_6_ | 308.290 | C_14_H_16_N_2_O_6_ | 308.290 | 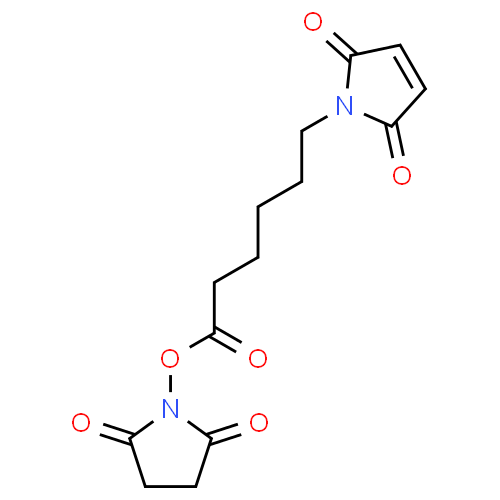 |
| GMBS | No | C_12_H_12_N_2_O_6_ | 280.236 | C_12_H_12_N_2_O_6_ | 280.236 | 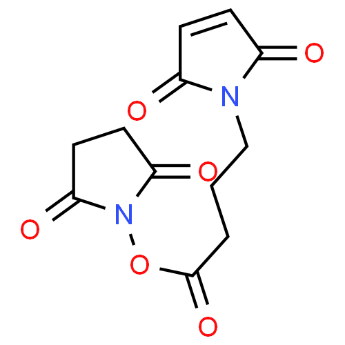 |
| LC-SMCC | No | C_22_H_29_N_3_O_7_ | 447.488 | C_22_H_29_N_3_O_7_ | 447.488 | 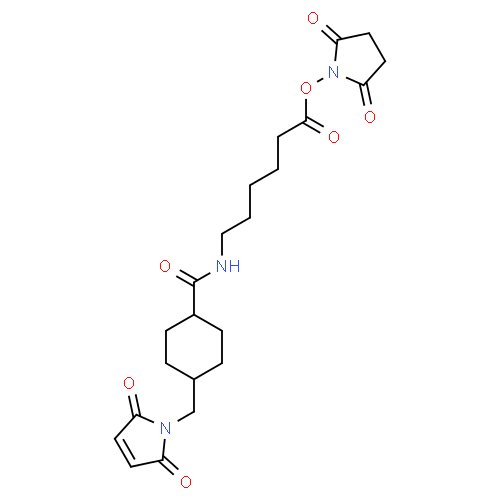 |
| LC-SPDP | Yes | C_18_H_23_N_3_O_5_S_2_ | 425.530 | C_13_H_19_N_2_O_5_S  C_5_H_4_NS | 315.370  110.160 | 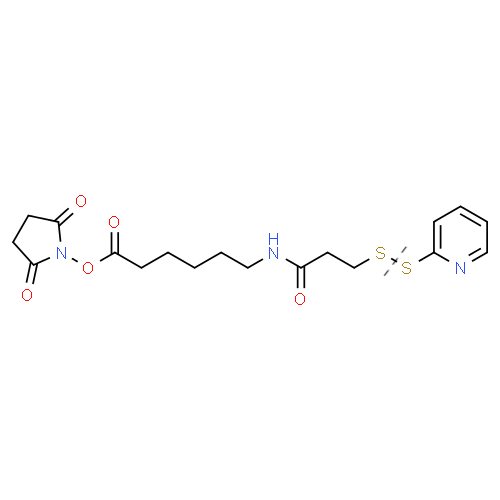 |
| MBS | No | C_15_H_10_N_2_O_6_ | 314.253 | C_15_H_10_N_2_O_6_ | 314.253 | 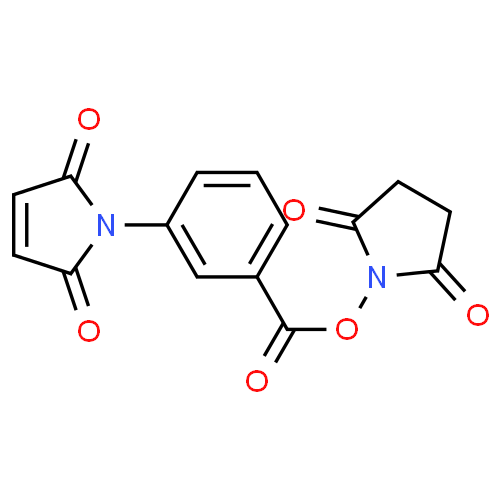 |
| PEG12-SPDP | Yes | C_39_H_65_N_3_O_17_S_2_ | 912.085 | C_5_H_4_NS  C_34_H_61_N_2_O_17_S | 110.160  801.925 | 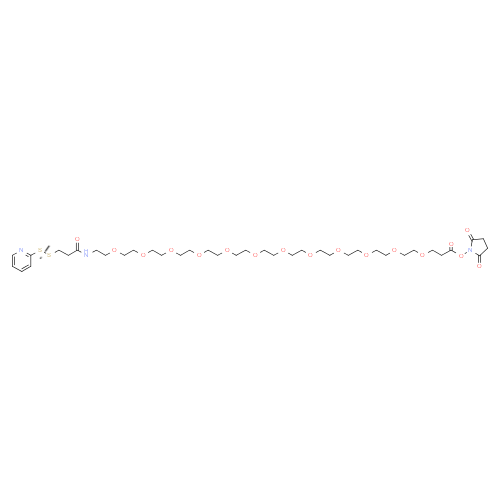 |
| PEG4-SPDP | Yes | C_23_H_33_N_3_O_9_S_2_ | 559.661 | C_5_H_4_NS  C_18_H_29_N_2_O_9_S | 110.160  449.501 | 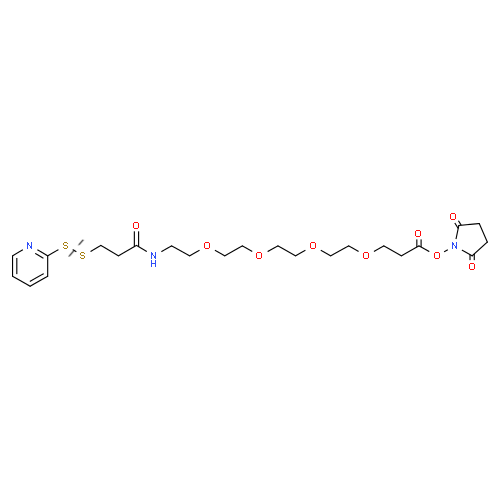 |
| SBAP | No | C_9_H_11_N_2_O_5_Br | 307.100 | C_9_H_11_N_2_O_5_Br | 307.100 | 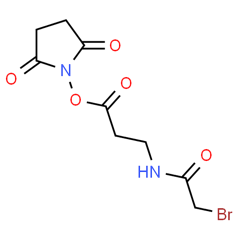 |
| SIA | No | C_6_H_6_INO_4_ | 283.021 | C_6_H_6_INO_4_ | 283.021 | 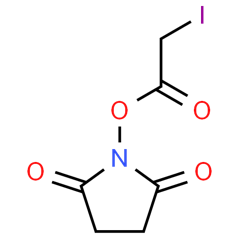 |
| SIAB | No | C_13_H_11_IN_2_O_5_ | 402.144 | C_13_H_11_IN_2_O_5_ | 402.144 | 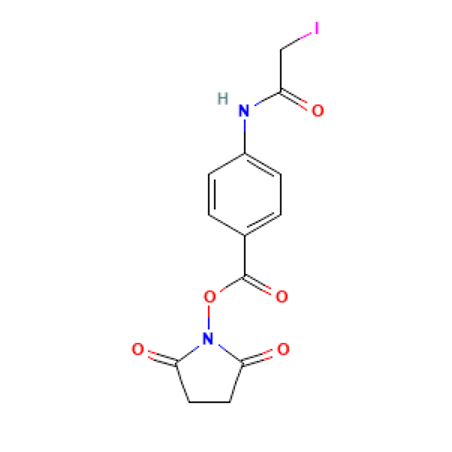 |
| SM(PEG)12 | No | C_38_H_63_N_3_O_19_ | 865.924 | C_38_H_63_N_3_O_19_ | 865.924 | 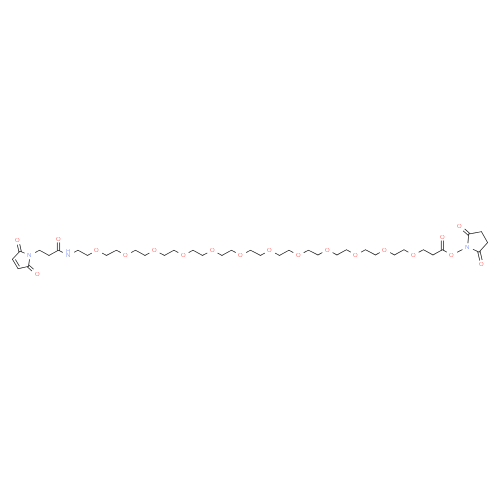 |
| SM(PEG)2 | No | C_18_H_23_N_3_O_9_ | 425.394 | C_18_H_23_N_3_O_9_ | 425.394 | 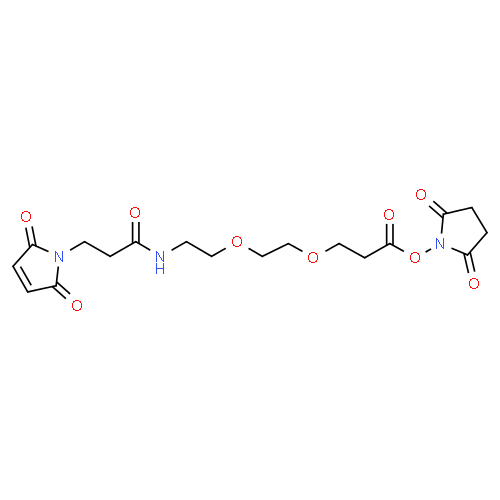 |
| SM(PEG)24 | No | C_62_H_111_N_3_O_31_ | 1394.560 | C_62_H_111_N_3_O_31_ | 1394.560 | 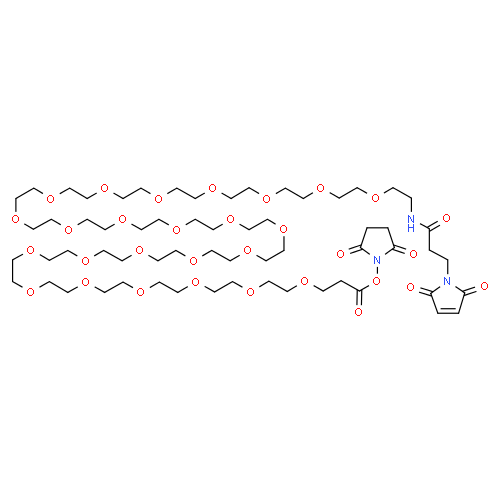 |
| SM(PEG)4 | No | C_22_H_31_N_3_O_11_ | 513.500 | C_22_H_31_N_3_O_11_ | 513.500 | 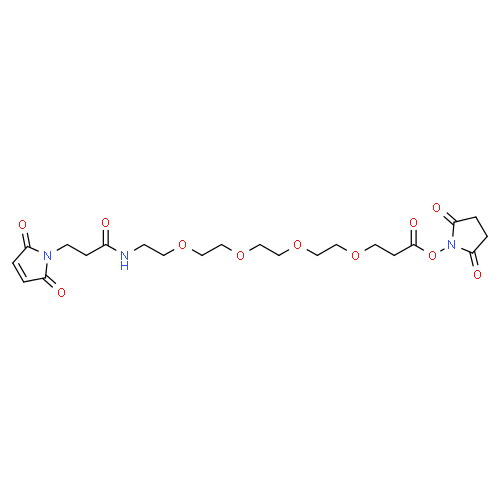 |
| SM(PEG)6 | No | C_26_H_39_N_3_O_13_ | 601.606 | C_26_H_39_N_3_O_13_ | 601.606 | 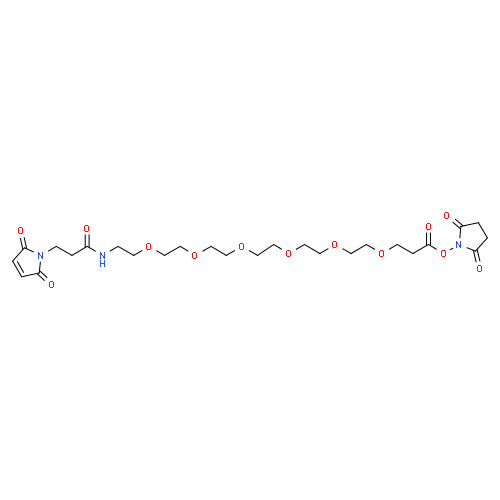 |
| SM(PEG)8 | No | C_30_H_47_N_3_O_15_ | 689.712 | C_30_H_47_N_3_O_15_ | 689.712 | 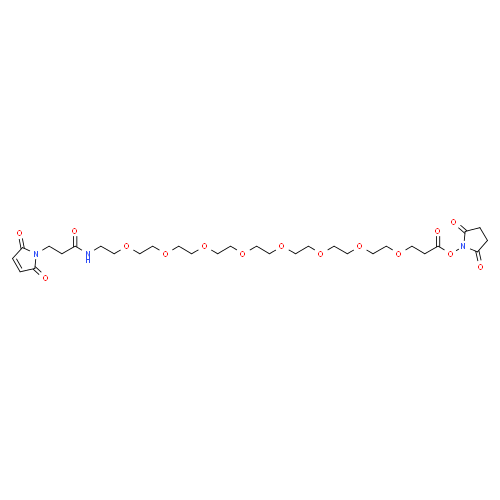 |
| SMCC | No | C_16_H_18_N_2_O_6_ | 334.328 | C_16_H_18_N_2_O_6_ | 334.328 | 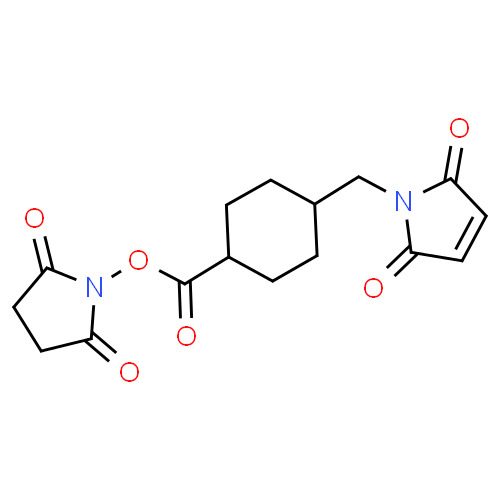 |
| SMPB | No | C_18_H_16_N_2_O_6_ | 356.334 | C_18_H_16_N_2_O_6_ | 356.334 | 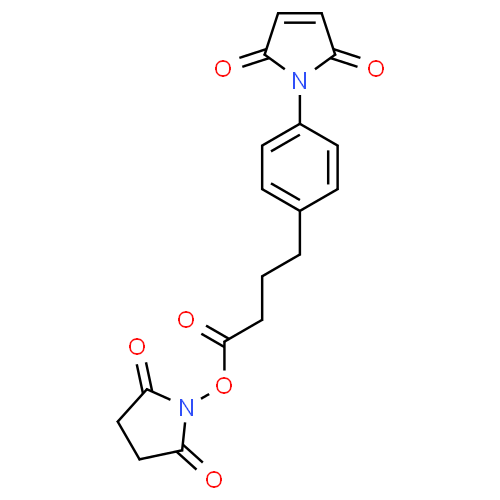 |
| SMPH | No | C_17_H_21_N_3_O_7_ | 379.369 | C_17_H_21_N_3_O_7_ | 379.369 | 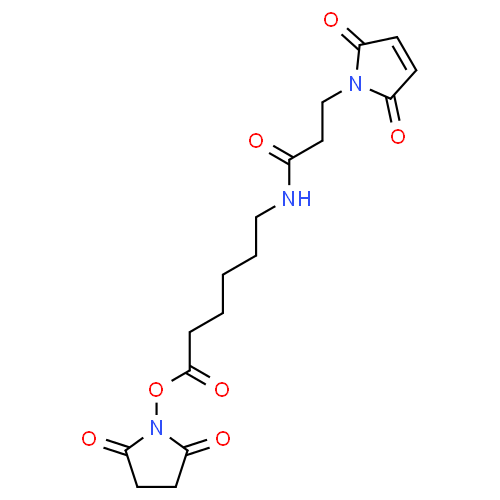 |
| SMPT | Yes | C_18_H_16_N_2_O_4_S_2_ | 388.468 | C_5_H_4_NS  C_13_H_12_NO_4_S | 110.160  278.308 | 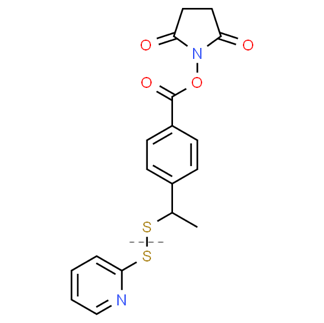 |
| SPDP | Yes | C_12_H_12_N_2_O_4_S_2_ | 312.370 | C_5_H_4_NS  C_7_H_8_NO_4_S | 110.160  202.210 | 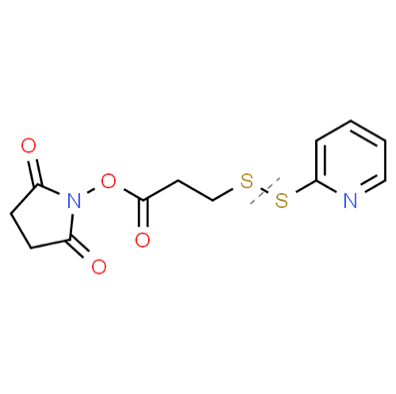 |
| Sulfo-EMCS | No | C_14_H_15_N_2_NaO_9_S | 410.335 | C_14_H_15_N_2_NaO_9_S | 410.335 | 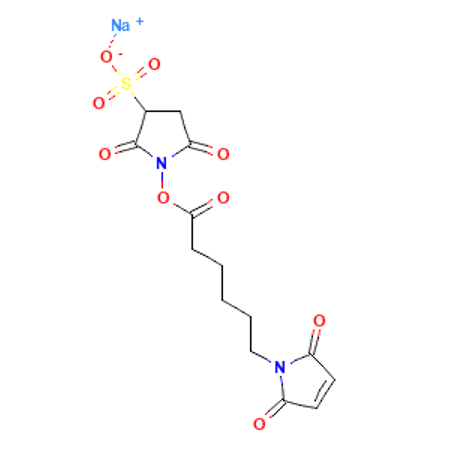 |
| Sulfo-GMBS | No | C_12_H_11_N_2_NaO_9_S | 382.281 | C_12_H_11_N_2_NaO_9_S | 382.281 | 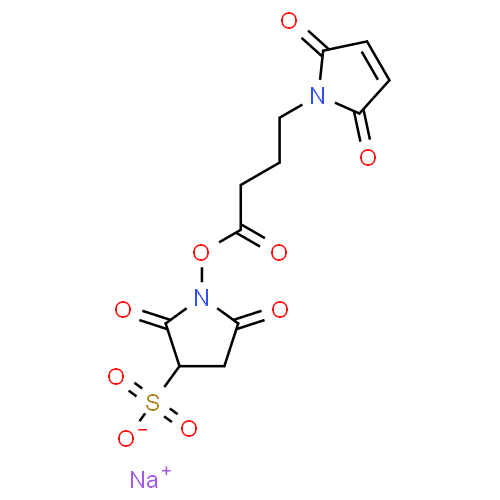 |
| Sulfo-KMUS | No | C_19_H_25_N_2_NaO_9_S | 480.470 | C_19_H_25_N_2_NaO_9_S | 480.470 | 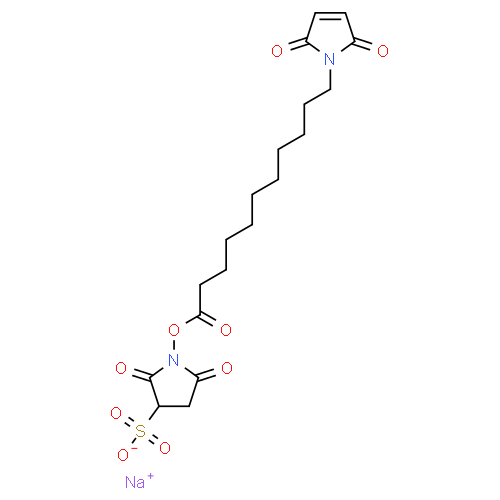 |
| Sulfo-LC-SPDP | Yes | C_18_H_22_N_3_NaO_8_S_3_ | 527.575 | C_13_H_18_N_2_NaO_8_S_2_  C_5_H_4_NS | 417.415  110.160 | 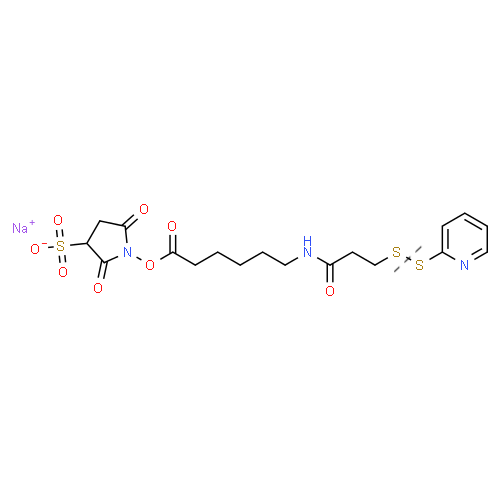 |
| Sulfo-MBS | No | C_15_H_9_N_2_NaO_9_S | 416.298 | C_15_H_9_N_2_NaO_9_S | 416.298 | 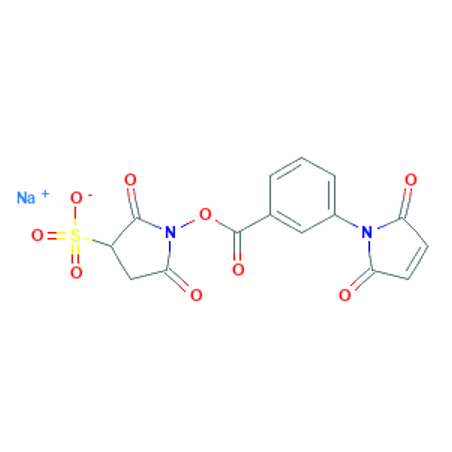 |
| Sulfo-SIAB | No | C_13_H_10_IN_2_NaO_8_S | 504.189 | C_13_H_10_IN_2_NaO_8_S | 504.189 | 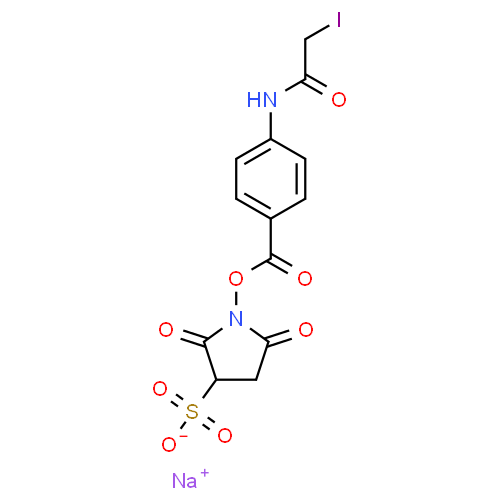 |
| Sulfo-SMCC | No | C_16_H_17_N_2_NaO_9_S | 436.373 | C_16_H_17_N_2_NaO_9_S | 436.373 | 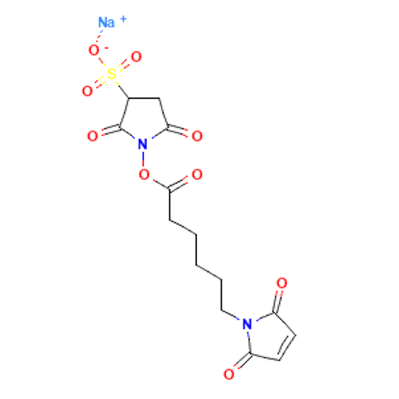 |
| Sulfo-SMPB | No | C_18_H_15_N_2_NaO_9_S | 458.379 | C_18_H_15_N_2_NaO_9_S | 458.379 | 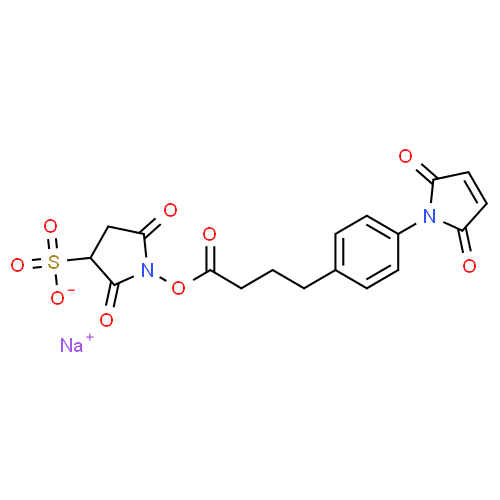 |
| IA-Alkyne | No | C_8_H_12_INO | 223.013 | C_8_H_12_INO | 223.013 | 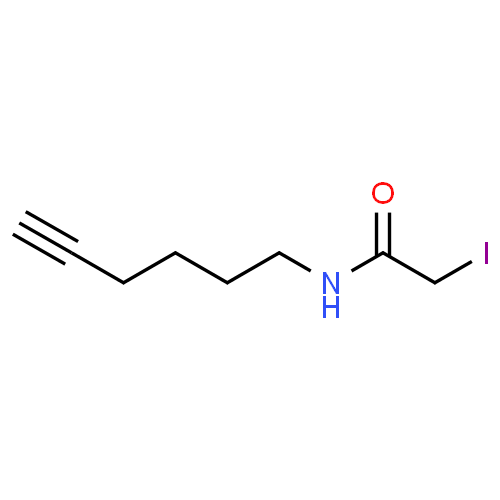 |
| DBCO-PEG4-maleimide | No | C_36_H_42_N_4_O_9_ | 674.751 | C_36_H_42_N_4_O_9_ | 674.751 | 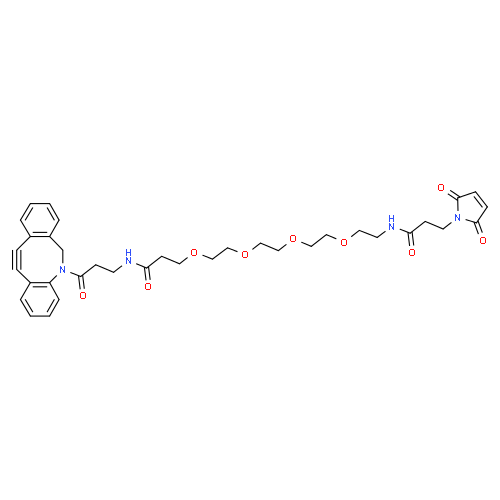 |
| MPBH | No | C_14_H_16_ClN_3_O_3_ | 309.753 | C_14_H_16_ClN_3_O_3_ | 309.753 | 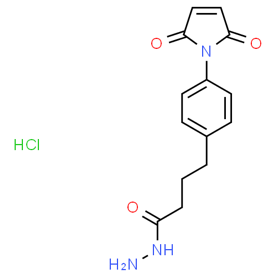 |
| PDPH | Yes | C_8_N_3_S_2_OH_11_ | 229.328 | C_5_NSH_4_  C_3_N_2_SOH_7_ | 110.160  119.168 | 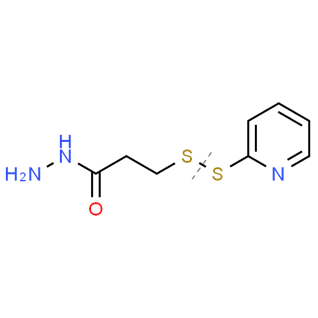 |
| PMPI | No | C_11_H_6_N_2_O_3_ | 214.180 | C_11_H_6_N_2_O_3_ | 214.180 | 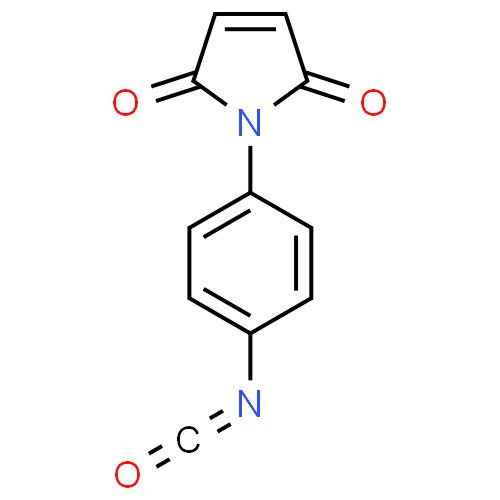 |
| BM(PEG)2 | No | C_14_H_16_N_2_O_6_ | 308.290 | C_14_H_16_N_2_O_6_ | 308.290 | 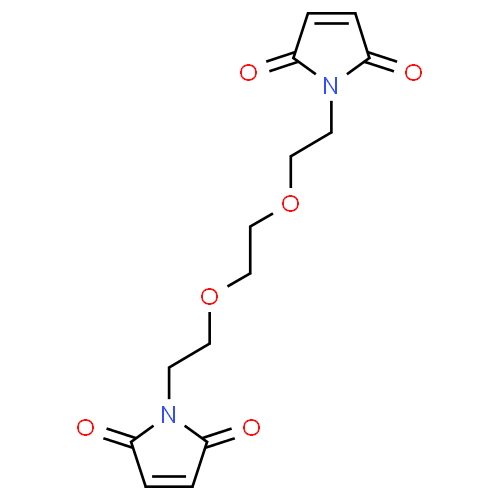 |
| BM(PEG)3 | No | C_16_H_20_N_2_O_7_ | 352.343 | C_16_H_20_N_2_O_7_ | 352.343 | 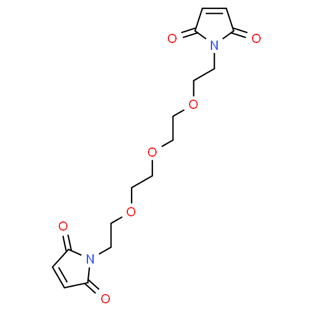 |
| BMB | No | C_12_H_12_N_2_O_4_ | 248.238 | C_12_H_12_N_2_O_4_ | 248.238 | 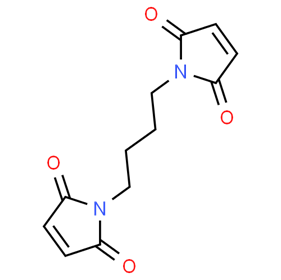 |
| BMH | No | C_14_H_16_N_2_O_4_ | 276.292 | C_14_H_16_N_2_O_4_ | 276.292 | 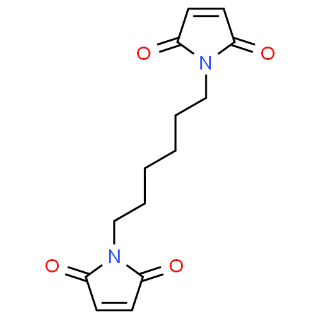 |
| BMOE | No | C_10_H_8_N_2_O_4_ | 220.184 | C_10_H_8_N_2_O_4_ | 220.184 | 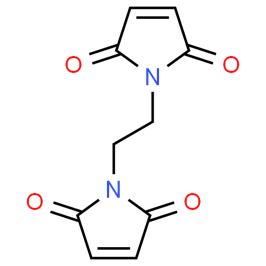 |
| DTME | Yes | C_12_H_12_N_2_S_2_O_4_ | 312.370 | C_6_H_6_NSO_2_  C_6_H_6_NSO_2_ | 156.185  156.185 | 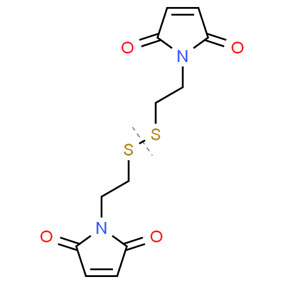 |
| TMEA | No | C_18_H_18_N_4_O_6_ | 386.364 | C_18_H_18_N_4_O_6_ | 386.364 | 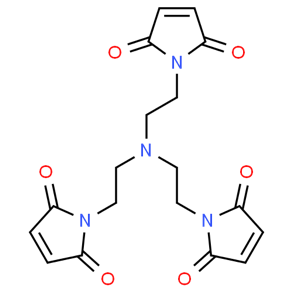 |
| SBA | No | C_6_H_6_BrNO_4_ | 236.021 | C_6_H_6_BrNO_4_ | 236.021 | 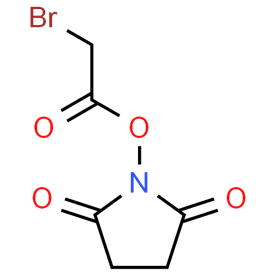 |
| DSSeb | No | C_18_H_24_N_2_O_8_ | 396.396 | C_18_H_24_N_2_O_8_ | 396.396 | 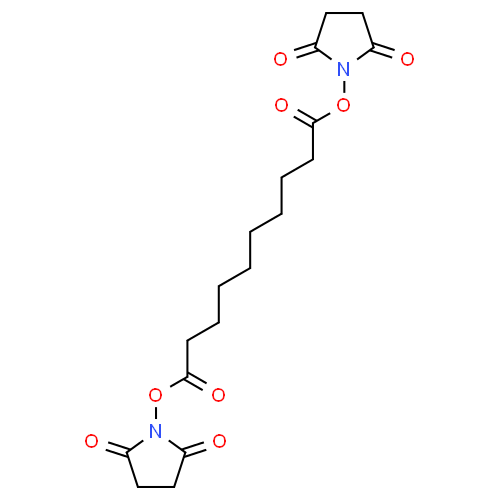 |
| Sulfo-SIA | No | C_6_H_7_INO_7_S | 364.092 | C_6_H_7_INO_7_S | 364.092 | 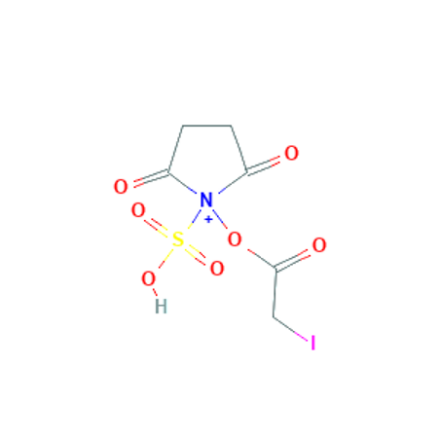 |
| SPDB | Yes | C_13_H_14_N_2_O_4_S_2_ | 326.397 | C_8_H_10_NO_4_S  C_5_H_4_NS | 216.237  110.160 | 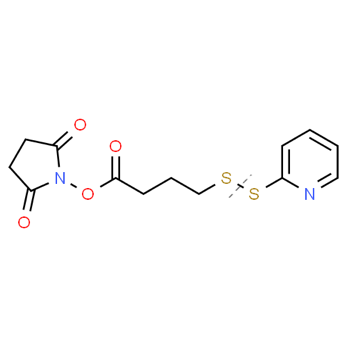 |
